# Driving Behaviors in Iran: A Descriptive Study Among Drivers of Mashhad City in 2014

**DOI:** 10.5539/gjhs.v7n7p39

**Published:** 2015-03-26

**Authors:** Mojtaba Mousavi Bazzaz, Ahmadreza Zarifian, Maryam Emadzadeh, Veda Vakili

**Affiliations:** 1 Department of Community Medicine, Mashhad University of Medical Sciences (MUMS), Mashhad, Iran; 2 Student Research Committee, Mashhad University of Medical Sciences (MUMS), Mashhad, Iran

**Keywords:** driving behavior, mashhad, risk factors, driving behavior questionnaire

## Abstract

**Background:**

Driver-related behaviors are substantial causes for motor vehicle accidents. It has been estimated that about 95% of all accidents are due to driver-related dangerous behaviors and approximately 60% of accidents are directly caused by driving behaviors. The aim of this study was to assess driving behaviors and its possible related factors among drivers in Mashhad city, Iran.

**Method:**

In a cross-sectional design, a total number of 514 drivers in Mashhad, Iran Surveyed. Manchester driver behavior questionnaire with 50 questions evaluated dangerous driving behaviors in 4 categories “aggressive violations”, “ordinary violations”, “errors” and “lapses”.

**Results:**

In this study, the median age of drivers was 31. Besides, 58.2% of men mentioned having a history of driving accident. Our study indicated smoking and alcohol drinking as risk factors of having more accidents. Hookah abuse is a predictor of aggressive violations and errors.

**Conclusion:**

This is the first study to assess the relation of personal car and its market value with the likelihood of having accidents. Due to major influences of driving fines, cigarette smoking, alcohol consumption and addiction on violations and errors, we recommend pivotal measures to be taken by road safety practitioners regarding driving surveillance.

## 1. Introduction

Accidents are among the most important challenges for community health, causing physical and mental damages and financial problems. Traffic collisions are predicted to become the third leading cause of disability adjusted life years (DALYs) lost by 2020 (Peden, McGee, & Krug, 2002). Crashes of automobiles, are major causes of deaths of people between 4 and 34 years old. The rate is highest for new drivers during the first few months driving on their own (National Research Council (NRC) et al., 2007). Since the most vulnerable age group are young adults, accidents impose an economic burden on the society (Elvik, 2000; Peden, 2004). Road accidents, with 1.3 million deaths per year, ranked ninth among the leading causes of death in 2004 and are estimated to climb the ranking ladder up to fifth by 2030 (Mathers, Fat, & Boerma, 2008). Injuries caused by road accidents are predictable and preventable causes of social damages, which can be prevented up to 98% (Park, 2007; Sharma, 2008).

It is estimated that more than 85% of deaths and 90% of life years lost from road accidents are happening in developing countries (Nantulya & Reich, 2002). According to the reports of World Health Organization (2010), with 34.1 road traffic deaths per 100,000 population in each year, Iran has been ranked the fifth worldwide and the first among Eastern Mediterranean countries, regarding road traffic deaths (World Health Organization [WHO], 2010). Approximately 7.5% of all national deaths in Iran are caused by road traffic injuries, which is the highest in the region (Akbari, Naghavi, & Soori, 2006).

The principal cause in 60% of all road traffic accidents is driver-related behaviors (Evans, 1996). It has been estimated that about 90-95% of all accidents occur due to driver-related dangerous behaviors (Rumar, 1985). So the aim of this study was to assess driving behaviors and its possibly related factors among drivers in Mashhad city, Iran in 2014.

## 2. Methods

Mashhad is the second most populous city in Iran and is the capital of Razavi Khorasan Province. Its population was 2,772,287 at the 2011 population census. The city is however most well-known and respected for housing the shrine of Imam Reza, the eighth Shia Imam. Every year millions of pilgrims visit the holy shrine. In this cross-sectional study, we surveyed a total number of 514 drivers in Mashhad, Iran. Survey was done using the Manchester driver behavior questionnaire (DBQ) (Lawton et al., 1997; Parker et al., 1998), which consists of 50 questions evaluating dangerous driving behaviors and categorizing them into four groups of “aggressive violations”, “ordinary violations”, “errors” and “lapses”. DBQ is one of the most widely used instruments in Traffic Psychology for measuring self-reported driving style and investigating the relationship between driving behaviour and accident involvement ((Dde Winter and Dodou, 2010, report 174 studies using some version of the DBQ).

We used a Persian version of the questionnaire, being valid and reliable before (Arizi & Haghayegh, 2009). Demographic information, including age, sex, academic degree, job status (in terms of income), history of smoking as well as drug or alcohol abuse, driving accident history since getting the driving license and number of driving fines were also included in the questionnaire and related to driving behaviors. Data collection was done referring to taxi stations, automobile agencies, public parking lots and car parks of shopping centers, banks, hospitals and universities all around the city. Parking lots of Imam Reza holy shrine were also places for sample collection. A total number of 514 questionnaires were completed by randomly selected drivers. We intended to cover all social classes, so data were collected from different parts of the city and tried to match our sample size with our geographical coverage. Verbal informed consent was obtained from participants. Ethics Committee of Mashhad University of Medical Sciences approved the study.

Statistical package for social sciences (SPSS) 11.5 software (SPSS Inc., Chicago, Illinois, USA) was used for all statistical analyses. Standard descriptive statistics were applied to describe the pattern of the data. Chi-square test was used to examine the significance of the association between categorical data. Normality of the data was checked with Kolmogorov–Smirnov test.

Kruskal-Wallis test was applied in non-normal distributions. Linear regression tests were used to predict “aggressive violations”, “ordinary violations”, “errors” and “lapses”. All tests were 2-tailed, and probability values below 0.05 were considered as statistically significant.

## 3. Results

More than 500 drivers participated in this cross-sectional study. The youngest driver was 18 and the oldest one was 85. The median age was 31. Table 1 shows demographic characteristics of all participants. In this study, 58.2% of men confessed having a driving accident previously.

**Table 1:** Demographic characteristics of study participants according to number of driving accidents

		Accident times during recent two years			P-value

		Never	1 or 2 times	3 times& more	
Age(mean±SD)		33.98±11.48	35.88±12.86	36.25±12.77	0.37*
Sex (N (%))	Female	28(34.1)	41(50)	13(15.9)	0.61
	male	100(39.2)	112(43.9)	43(16.9)	
Marital status (N (%))	Single	46(41.4)	48(43.2)	17(15.3)	0.62
	Married	81(36.2)	103(46)	40(17.9)	
Job status (N (%))	Paid job	90(41.1)	94(42.9)	35(16)	0.21
	Non paid job	37(31.4)	59(50)	22(18.6)	
Education (N (%))	Less than diploma	25(36.8)	32(47.1)	11(16.2)	0.91
	Diploma & higher	98(37.8)	115(44.4)	46(17.8)	
Residential place (N (%))	High class	29(31.9)	46(50.5)	16(17.5)	0.94
	Low class	35(34)	51(49.5)	17(16.5)	
Driving license(mean±SD)		10.98±10.18	12.53±10.82	11.24±7.81	0.38*
Cigarette Smoking (N (%))		26(29.9)	41(47.1)	20(23)	0.10
Addiction (N (%))		3(21.4)	10(71.4)	1(7.1)	0.12
Alcohol (N (%))		14(41.2)	12(35.3)	8(23.5)	0.38
Hookah Smoking (N (%))		43(37.4)	49(42.6)	23(20)	0.54
Personally automobile (N (%))		94(35.2)	121(45.3)	52(19.5)	0.01
Car price (N (%))	Less than 6000 USD	65(39.6)	67(40.9)	32(19.5)	0.02**
	6000-15000 USD	43(32.3)	70(52.6)	20(15)	
	15000-30000 USD	1(6.7)	9(60)	5(33.3)	
	≥30000 USD	6(60)	4(40)	0(0)	
Time (N (%))	night	54(36.5)	67(45.3)	27(18.2)	0.88
	Other time	70(38)	84(45.7)	30(16.3)	
Stress (N (%))		27(31)	39(44.8)	21(24.1)	0.04
Anger (N (%))		48(37.5)	54(42.2)	26(20.3)	0.29
Penalty(mean±SD)		2.27±3.06	4.58±6.16	7.43±7.18	<0.001*
Route (N (%))	Highway	56(41.2)	59(43.4)	21(15.4)	0.62
	Ordinary way	68(36.2)	86(45.7)	34(18.1)	

* Kruskall-Wallis Test;

** Fisher’s Exact Test.

We categorized our study subjects, in terms of accident history into three groups: accident free group, those who had less than three accidents, and those with a history of three accidents or more. As mentioned in table 1, 76.7% of people with a history of more than two accidents, were males. Mean age in this group was 36.25 years. 41.1% of those with a paid job did not have an accident in recent years, but this rate has fallen to 31.4% in drivers without a paid job. Eighty percent of those with more than two accidents had at least high school diploma.

The numbers of addicted and alcoholic drivers were 22 and 41 respectively. Among alcohol consumers, 23.5% had a history of more than two accidents, while 16.5% of non-alcoholic people reported this history. Twenty-three percent of smokers and fourteen percent of non-smokers had the history of three accidents and more.

About 75% of accident-free group had personal car, while this rate increased to 95% in those with the history of more than two accidents. Drivers owning a personal car were significantly less likely to have accidents (P-value = 0.01) and car price had a significant difference among three groups (P-value = 0.02) (Table 1).

31% of stressful people and 37.5% of furious people (according to self-reports of stress and anger) did not have any accident during their driving life. There was no significant difference between the time of driving (day or night) and number of accidents (Table 1). 63.8% of those who chose ordinary ways and encountered heavier traffics had at least one accident, while this rate declined to 58.8% for those who mostly chose highways. Table 2 depicts the detail of driving behaviors among study participants in four dimensions including “aggressive violations”, “ordinary violations”, “errors” and “lapses”. Table 3 shows the predictors for each driving behavior.

**Table 2:** Risky driving behavior in study participants according to sociodemographic factors

			Lapses	Aggressive violation	Ordinary violation	Error
Age (r)*			-0.074	-0.144	-0.068	-0.013
		p-value	0.11	0.002	0.14	0.78
Fine (r)*			0.019	0.046	0.048	0.036
		p-value	0.72	0.38	0.36	0.49
Marital status**(mean±SD)	Single		20.39±12.03	18.04±11.21	3.06±2.13	7.93±5.55
	Married		19.13±10.05	16.64±10.44	2.83±2.03	7.75±4.7
		p-value	P= 0.22	0.11	0.22	0.92
Education**(mean±SD)	Less than diploma		20.01±10.72	16.55±10.07	2.65±2.07	8.3±4.98
	Diploma and higher		19.65±11.04	17.39±10.96	2.98±2.06	7.82±5.12
		p-value	0.69	0.53	0.13	0.31
Driving License (r)*			-0.179	-0.146	-0.116	-0.126
		p-value	<0.001	0.002	0.016	0.009
Residential place**(mean±SD)	High class		19.03±9.84	17.17±11.08	2.88±1.75	7.9±4.77
	Low class		18.75±10.08	16.51±10.4	2.94±2.17	7.25±4.71
		p-value	0.78	0.61	0.85	0.14
Route**(mean±SD)	Highway		19.3±11.79	18.25±11.36	2.92±2	7.76±5.65
	Ordinary way		19.94±10.85	16.58±10.69	2.94±2.13	7.94±4.96
		p-value	0.29	0.1	0.96	0.32
Time**(mean±SD)	night		21.02±10.61	19.26±11.56	3.13±1.88	8.45±5.04
	Other time		18.56±11.44	5.65±10.17	2.74±2.19	7.41±5.28
		p-value	<0.001	<0.001	0.006	0.002
Cigarette smoking*			-0.222	-0.323	-0.172	-0.236
		p-value	<0.001	<0.001	<0.001	<0.001
Car price*			0.001	0.044	0.06	0.045
		p-value	0.97	0.34	0.194	0.32

* Pearson Correlation;

** Mann-Whitney Test.

**Table 3:** Predictors of risky driving behavior- linear regression

		Unstandardized coefficient		Standardized coefficient		
	
		B	Standard Error	Beta	t	Sig
Lapses	Constant	28.662	3.927		7.298	<0.001
	Cigarette smoking	-5.341	1.971	-2.710	-2.710	0.008
	Driving license	-0.183	0.074	-0.199	-2.462	0.015
	accident	0.951	0.453	0.170	2.100	0.038
Aggressive violation	Constant	40.314	4.389		9.184	<0.001
	Hookah smoking	-5.296	2	-0.244	-2.647	0.009
	Age	-0.154	0.068	-0.180	-2.264	0.025
	Cigarette smoking	-5.319	2.456	-0.194	-2.165	0.032
Ordinary violation	Constant	3.273	0.903		3.626	<0.001
	Cigarette smoking	-1.002	0.405	-0.207	-2.474	0.015
	Job status	1.184	0.547	0.181	2.162	0.032
Error	Constant	10.508	1.762		5.964	<0.001
	Hookah smoking	-2.221	0.714	-0.249	-3.113	.002
	Accident location	1.927	0.687	0.227	2.804	.006
	Residential place	-1.864	0.668	-0.226	-2.789	.006

Based on our results, smoking has a major role in anticipating all dangerous driving behaviors, except errors. However, hookah is a predictor for Aggressive violations and Errors. Drivers’ age and job are predictors of aggressive and ordinary violations, respectively. Table 3 depicts predictors of dangerous driving behaviors.

## 4. Discussion

Driving is a complicated behavior, which is related to many underlying factors. Thus, we used DBQ, which assess and categorize risky driving behaviors we divided illegal and dangerous behaviors into two major categories of violations and errors, which are in line with previous studies (Lajunen, Parker, & Summala, 2004; Reason, Manstead, Stradling, Baxter, & Campbell, 1990).

Literature suggests that our study is the first of its kind to assess the relation of owning a personal car and car price with the likelihood of having accidents. The probability of having accident has been outstandingly decreased among drivers who owned cars costing more than 30000 USD. On one hand, this could be due to their cautious driving behaviors to avoid car damage and financial loss. On the other hand, it may be an incorrect conclusion because of small number of participants in this group. Hence, more research is needed in this group.

Although our results showed that self-reports of anger and excitement-seeking were not significantly related to history of road accidents, many previous studies have shown a significantly higher probability of having accident in angry drivers (Hagh-Shenas, Hosseini, Jamshidi, & Azizi, 2008; Nabi et al., 2005). Staicu and his colleagues also found that anger can be the cause of growing number of car accidents (Staicu & Cuțov, 2010).

Results of this study showed that stressful and anxious drivers were significantly less prone to accidents. This may be due to our different means in measuring anger and stress, whereas in the present study we relied only on self-reports of anger and anxiety, which may be an inaccurate tool for measuring these emotions. Many previous studies have shown the effect of personality traits on driving behavior (Beanland, Sellbom, & Johnson, 2014; Hagh-Shenas et al., 2008).

Three groups of drivers in this study did not show any significant difference regarding alcohol consumption, addiction, and cigarette smoking, probably due to low prevalence of cigarette smoking, addiction, and alcohol abuse in the city. However, many studies found significantly higher rate of accidents among alcohol-dependent, smokers, and addicted drivers (Jakubczyk et al., 2013; Mangiaracina & Palumbo, 2007; Reece, 2008).

This study had a few limitations. Since our main goal was not measuring stress, anger, and excitement in drivers, we have only received self-reports, which could influence our results. The other limitation is that we did not separate smokers into two groups who smoke while driving and those who do not smoke while driving.

In this study, age was significantly related with aggressive violations, while history of fines, cigarette smoking, time of driving and years past driving license were significantly related with all four risky driving behaviors.

In a meta-analysis deWinter and Dodou, assessed the relation of violations and errors from the DBQ to accident involvement. They indicated younger age to be positively associated with violations, which is probably due to confidence building in beginner drivers. On the other hand, they mentioned a negative association between younger age and errors, which might be because of the learning process in these novice drivers and consequent error reduction. They also mentioned male and female gender as predictors of violation and errors, respectively (De Winter & Dodou, 2010).

Our results indicate that the strongest predictor of accident among drivers is history of driving fines, while traffic violation was the most common and the main cause of road traffic injuries in previous studies in other countries (Bener, 2012; Pourabdian & Azmoon, 2013).

In conclusion, we suggest road safety practitioners to focus on factors such as driving fines, which is significantly associated with reduction in violations and errors, in constructing surveillance systems. Indubitably, more strict driving surveillances leads to less risky driving behaviors and road accidents.

## References

[ref1] Akbari M, Naghavi M, Soori H (2006). Epidemiology of Deaths from Injuries in the Islamic Republic of Iran. Eastern Mediterranean Health Journal.

[ref2] Arizi H, Haghayegh S. A (2009). Psychometric Characteristics of Manchester Driver Behavior Questionnaire. Paayesh.

[ref3] Beanland V, Sellbom M, Johnson A. K (2014). Personality Domains and Traits that Predict Self-Reported Aberrant Driving Behaviours in a Southeastern US University Sample. Accident Analysis & Prevention.

[ref4] Bener A (2012). A Study on Road Traffic Crashes and Injuries in Qatar as Reported by Drivers. J Egypt Public Health Assoc.

[ref5] De Winter J, Dodou D (2010). The Driver Behaviour Questionnaire as a Predictor of Accidents: A Meta-Analysis. Journal of safety research.

[ref6] Elvik R (2000). How Much do Road Accidents Cost the National Economy?. Accident Analysis & Prevention.

[ref7] Evans L (1996). The Dominant Role of Driver Behavior in Traffic Safety. American Journal of Public Health.

[ref8] Hagh-Shenas H, Hosseini M, Jamshidi M, Azizi H. R (2008). Relation of Personality Traits with Driving Behavior in City of Shiraz in 2005. Hakim Research Journal.

[ref9] Jakubczyk A, Klimkiewicz A, Wnorowska A, Mika K, Bugaj M, Podgórska A, Wojnar M (2013). Impulsivity, Risky Behaviors and Accidents in Alcohol-Dependent Patients. Accident Analysis & Prevention.

[ref10] Lajunen T, Parker D, Summala H (2004). The Manchester Driver Behaviour Questionnaire: a Cross-Cultural Study. Accident Analysis & Prevention.

[ref11] Mangiaracina G, Palumbo L (2007). Smoking while Driving and its Consequences on Road Safety. Ann Ig.

[ref12] Mathers C, Fat D. M, Boerma J (2008). The global burden of disease.

[ref13] Nabi H, Consoli S. M, Chastang J. F, Chiron M, Lafont S, Lagarde E (2005). Type A Behavior Pattern, Risky Driving Behaviors, and Serious Road Traffic Accidents: a Prospective Study of the GAZEL Cohort. Am J Epidemiol.

[ref14] Nantulya V. M, Reich M. R (2002). The Neglected Epidemic: Road Traffic Injuries in Developing Countries. BMJ: British Medical Journal.

[ref15] National Research Council (US), Institute of Medicine (US), and Transportation Research Board (US) Program Committee for a Workshop on Contributions from the Behavioral and Social Sciences in Reducing and Preventing Teen Motor Crashes.

[ref16] Park K (2007). Park’s textbook of preventive and social medicine.

[ref17] Peden M (2004). World Report on Road Traffic Injury Prevention.

[ref18] Pourabdian S, Azmoon H (2013). The Relationship between Trait Anxiety and Driving Behavior with Regard to Self-reported Iranian Accident Involving Drivers. Int J Prev Med.

[ref19] Peden M, McGee K, Krug E (2002). Injury: a leading cause of the global burden of disease.

[ref20] Reason J, Manstead A, Stradling S, Baxter J, Campbell K (1990). Errors and Violations on the Roads: a Real Distinction?. Ergonomics.

[ref21] Reece A. S (2008). Experience of Road and other Trauma by the Opiate Dependent Patient: a Survey Report. Subst Abuse Treat Prev Policy.

[ref22] Rumar K (1985). The Role of Perceptual and Cognitive Filters in Observed Behavior. Human behavior and traffic safety.

[ref23] Sharma B (2008). Road Traffic Injuries: A Major Global Public Health Crisis. Public health.

[ref24] Staicu M.-L, Cuţov M (2010). Anger and Health Risk Behaviors. Journal of medicine and life.

[ref25] World Health Organization. Estimated road traffic death rate (per 100,000 population) (2010). http://gamapserver.who.int/gho/interactive_charts/road_safety/road_traffic_deaths2/atlas.html.

